# Evaluation of process parameters in the production of jasmonic acid

**DOI:** 10.1186/1753-6561-8-S4-P244

**Published:** 2014-10-01

**Authors:** Alexandre Zanelli dos Santos, Vera Lucia Araujo Leite, Murilo Daniel de Mello Innocentini, Miriam Verginia Lourenço

**Affiliations:** 1 Unidade de Biotecnologia, Universidade de Ribeirão Preto, UNAERP, Brazil; 2 Curso de Engenharia Química, Universidade de Ribeirão Preto, UNAERP, Brazil

## Introduction

The term jasmonates is used to describe lipid derivatives synthesized via octadecanoid pathway and are mainly represented by jasmonic acid (JA) and its ester methyl jasmonate (MJ). In plants, jasmonates act in the defense mechanism [1], and recent reports have attributed to JA and MJ the ability to inhibit the growth of human cancer cells, yet having cytotoxic selectivity [2]. Due to the diverse biological activities related to jasmonates, studies have been conducted to optimize the bioproduction route of these molecules, and the use of strains of *Botryosphaeria rhodina *seems to be a viable alternative when compared to the extraction from plants [3]. In order to optimize the production process, some of the important parameters must be standardized and controlled, such as the microorganism strain, the culture medium composition and the operational features of the process [4]. This study aimed to evaluate the influence of the inoculum size, medium supplementation with tryptone, the fermentation time and the interaction among these factors on the jasmonates production by the fungus *Botryosphaeria rhodina*.

## Methodology

The fermentations were performed using M2 culture medium with and without the addition of 5 g/L of tryptone. The volume of inoculum was 5 or 20 mL of homogenate (OD = 0.5 at λ = 700 nm), with average fermentation times of 5 and 10 days. Fermentations were conducted in the dark at 30°C under static conditions. The experiments were performed following a full 2^3 ^factorial design and performed in duplicate. For quantification of jasmonates produced at the end of the fermentation period, the fermented samples were recovered by vacuum filtration, the pH was adjusted to 3.0 with 4M HCl and then the liquid subjected to extraction with ethyl acetate. The jasmonates quantification was performed according to [1]. Based on AJ concentrations of each treatment, the variance of effects was calculated and T-test was applied at 5% significance level.

## Results and conclusion

From the measurements of JA in the fermented broth, the effects of each factor and their interactions were calculated. It was possible to verify that the JA production was significantly influenced (at 5% level) by the fermentation time, inoculum size, and the interaction between inoculum size and tryptone, so that the elevation of 5 to 10-day fermentation promoted an increase in the production of JA. The inoculum size factor presented a negative effect, namely, an increase of 5 to 20 ml of inoculum resulted in a lower production during JA fermentation. The factor medium supplementation with tryptone showed no significant effect; however there was a negative interaction between time and tryptone, ie, the addition of tryptone along time resulted in a lower production of JA. Thus the highest production of JA by the fungus *B. rhodina *under the tested conditions was 421.5 mg/L using the M2 culture medium without supplementation with tryptone, using 5 mL of homogenate for 10 days of fermentation.
